# Editorial: Exercise and Sport: Their Influences on Women's Health Across the Lifespan

**DOI:** 10.3389/fphys.2020.615468

**Published:** 2021-01-20

**Authors:** Cheryce L. Harrison, Angelica Lindén Hirschberg, Trine Moholdt

**Affiliations:** ^1^Monash Centre for Health Research and Implementation, School of Public Health and Preventive Medicine, Monash University, Clayton, VIC, Australia; ^2^Department of Women's and Children's Health, Karolinska Institutet, Stockholm, Sweden; ^3^Department of Gynecology and Reproductive Medicine, Karolinska University Hospital, Stockholm, Sweden; ^4^Department of Circulation and Medical Imaging, Norwegian University of Science and Technology, Trondheim, Norway; ^5^Women's Clinic, St. Olavs Hospital, Trondheim, Norway

**Keywords:** inflammation, contraceptives, adipose tissue, polycystic ovary syndrome, pregnancy, endurance training, strength training

This Research Topic of Frontiers in Physiology is dedicated to the memory of Professor Nigel Stepto, the Lead Guest Editor of this issue, who sadly passed away during its formation.

Prof. Stepto was a passionate and recognized world leader in the field of Exercise Physiology with outstanding contributions, particularly in the area of women's reproductive health. Nigel's research passion was in understanding the mechanistic effects of exercise for health and therapy with a special interest in insulin resistance and polycystic ovary syndrome, the leading cause of anovulatory infertility in young women of reproductive age. He was the co-Deputy Director—Research Training at the Institute of Health and Sport (IHeS) at Victoria University, Melbourne, Australia, and held adjunct associate professorial roles at Monash University and the University of Melbourne. He was Chair of the Exercise and Sports Science Association (ESSA) Research Committee, Project Director of the Australian Institute for Musculoskeletal Science (AIMSS) and an active member of the Australian Physiological Society (AuPS). Alongside his influential research career and leadership roles, Nigel was a strong advocate for post-graduate and early career researchers. His collaborative nature and approach to research ensured those mentored by him were considered, included and valued members across his many research projects and initiatives. Nigel's impact and influence on the careers of early researchers will continue at Victoria University with both a Nigel Stepto Travel Award and Nigel Stepto Ph.D. Scholarship established in his honor.

Nigel was great friend and colleague to many and is very much missed. Nigel is survived by his wife, Fiona and two children Matilda (14 years) and Harriet (11 years). Vale, Professor Nigel Stepto (12 September 1971−4 February 2020).

## Introduction

Physiological responses and adaptations to exercise that influence both health and sports performance is a broad and well-documented area of research with acute and prolonged effects now widely understood. Resultantly, exercise is recognized as a potent therapy for the prevention and treatment of chronic disease in adults.

Yet a significant research gender bias toward males remains with research elucidating differential reproductive and life-phase effects across clinical exercise, exercise and sports science in women, currently limited. This Research Topic was introduced to better explore physiological responses to exercise in women across the spectrum of health promotion to sports performance and the interplay of the reproductive lifespan on health and performance outcomes.

This Research Topic consists of nine articles, including six original research articles, two narrative review articles, and one systematic review and meta-analysis. A broad range of themes are covered across female exercise physiology, including the role of hormonal regulation and inflammation on exercise performance; the beneficial role of exercise in anovulatory conditions including polycystic ovary syndrome and perspectives of exercise during pregnancy and its role in hypertensive disorders.

## Training Adaptations Throughout the Female Lifespan

Fluctuation in ovarian hormone levels induces physiological alterations that can produce differences in exercise performance during the menstrual cycle (Janse de Jonge, 2003). For example, progesterone has been shown to increase ventilation and body temperature at rest (Marsh and Jenkins, 2002), whereas oestradiol modulates vascular flow (Joyner et al., 2015). Mandrup et al. investigated how menopausal status and high intensity exercise training influence adipose tissue mass, glucose uptake and protein content. They demonstrate similar improvements in cardiorespiratory fitness and decreases in subcutaneous and visceral adipose tissue mass following 3 months' training in pre- and post-menopausal women. They also report of similar insulin-stimulated glucose uptake in abdominal, gluteal, and femoral adipose tissue depots in pre- vs. post-menopausal women, in contrast to earlier findings from the same trial showering skeletal muscle insulin resistance in post- compared to premenopausal women (Mandrup et al., 2018).

High-repetition, low-load resistance training in group class settings has gained popularity for weight control, especially among women. One example of this type of training is BodyPump (Les Mills International), which is claimed to result in high energy expenditure. Rustaden et al. assessed energy expenditure during BodyPump compared with heavy load resistance exercise using indirect calorimetry in women who were overweight and found that both training modalities produced similar energy expenditure during and up to 140 min after the exercise session.

## Menstruation Cycle and Oral Contraceptives

Pereira et al. summarized the effects of the ovarian hormone fluctuations during the menstrual cycle on exercise-induced fatigability in a mini-review based on 46 studies comparing the follicular phase with the luteal phase of the menstrual cycle. In total, 15 studies demonstrated a statistical difference between the menstrual cycle phases studied. However, the results were inconsistent with seven studies reporting less fatigability during the luteal phase and eight studies reporting less fatigability during the follicular phase. The inconsistences could be explained by differences in exercise mode, the limb used, type of contraction and the classification of the menstrual cycle phase. The authors concluded that further studies are needed to determine the effects of a specific menstrual cycle phase on exercise-induced fatigability.

Exogenous hormones introduced through oral contraceptive use have also been found to influence exercise capacity (Lebrun et al., 2003) and change the metabolic, cardiovascular, and ventilatory responses to exercise (Charkoudian and Joyner, 2004; Isacco et al., 2012; Schaumberg et al., 2017). Schaumberg et al. observed a dampened response of central physiological adaptations to sprint interval training, demonstrated by pulmonary oxygen uptake kinetics, in women taking oral contraceptives compared to women with natural menstrual cycles. The potential underlying mechanisms for these observations include the influence of exogenous hormones on the overall endocrinological profile. Again, more knowledge is needed regarding the effect of oral contraceptives on exercise training adaptations.

In another study by Larsen et al., blood samples were collected from 53 elite female athletes prior to the Rio Olympic Games. The study showed that those who were taking oral contraceptives had higher levels of C-reactive protein (CRP) in the blood than those without hormonal contraception. CRP is a marker of inflammation and tissue damage, and it was suggested that this marker could have potential consequences for athlete performance and recovery. Other markers of stress and inflammation were comparable between groups.

## Polycystic Ovary Syndrome

Polycystic ovary syndrome (PCOS) is the most common endocrine disorder among women of reproductive age and characterized by anovulation, hyperandrogenism, and polycystic ovarian morphology (Charkoudian and Joyner, 2004). This condition is highly associated with reproductive, metabolic and mental complications. Insulin resistance and obesity are important etiological factors contributing to the severity of the symptoms. Lifestyle intervention, including exercise, is recommended as a first-choice therapy to improve health outcomes (Charkoudian and Joyner, 2004). However, it is unclear what kind of exercise is most effective. Patten et al. performed a systematic review and meta-analysis of exercise interventions in PCOS. Based on 19 articles and 777 women, it was demonstrated that exercise training improved cardiorespiratory fitness, body composition, and insulin resistance. It was also clear that improvements were dependent on exercise intensity rather than duration. The results suggest that a minimum of 120 min of vigorous intensity per week is needed to provide beneficial health outcomes for women with PCOS.

MicroRNAs (miRNAs) are small non-coding RNAs that regulate gene expression post-transcriptionally and have been suggested to be of importance for the pathophysiology of insulin resistance in PCOS. Lionett et al. studied circulating and adipose tissue miRNAs in women with PCOS compared to controls and in response to randomized low-volume or high-volume high intensity interval training. They found that women with PCOS have higher circulating levels of miRNA-27b compared to non-PCOS women but comparable adipose tissue miRNAs. miRNA-27b has been implicated in several metabolic and cellular processes, such as fatty acid metabolism, adipocyte differentiation and inflammation (Chen et al., 2012). In response to 16 weeks of low-, but not high-volume, high intensity training, levels of miRNA-27b were reduced. The clinical significance of these findings needs to be studied further.

## Pregnancy

European and American guidelines advocate that pregnant women should be physically active at least 150 min/week to optimize gestational weight gain and prevent adverse pregnancy outcomes, such as gestational diabetes and hypertensive disorders (2015; Gynaecologists RCoO, 2015). However, <15% of pregnant women adhere to these recommendations (Gjestland et al., 2013). This Research Topic contains a Mini Review (Witvrouwen et al.) on the effects of exercise training during pregnancy on vascular health, with a focus on gestational hypertensive disorders (gestational hypertension and pre-eclampsia). Conflicting reports were found with a need for further research to fully elucidate efficacy of exercise in reducing risk of hypertensive disorders during pregnancy. The largest systematic review [22 randomized controlled trials (RCTs), *n*= 5,316] and meta-analysis to date showed that exercise during pregnancy significantly reduced the risk of gestational hypertension (OR 0.61, 95% CI 0.43–0.85), as well as pre-eclampsia (OR 0.59, 95% CI 0.37–0.94) (15 RCTs, *n* = 3,322) (Davenport et al., 2018). However, five out of nine systematic reviews and meta-analyses showed no significant effect. The authors suggest that the discrepancies may be caused by different methodological issues. Yet given the benefits of regular physical activity on health and well-being generally, health care providers should be encouraged to discuss lifestyle behaviors with pregnant women to optimize maternal and child health outcomes. Health care provider perspectives on lifestyle advice, during pregnancy, weight gain, physical activity, and nutrition was explored by Haakstad et al. While most midwives viewed lifestyle counseling as important, nearly 40% did not give advice on gestational weight gain or gave advice discordant with the recommendations from the Institute of Medicine (Rasmussen and Yaktine, 2009), emphasizing the need for increased support and guidance for health professionals initiating healthy lifestyle conversations during pregnancy.

## Perspectives

The manuscripts included in this Research Topic contribute to much needed research into the role and influence of reproduction on physiological responses to exercise across the spectrum of female exercise physiology. Beneficial effects of exercise are reported underscoring its importance in women's health broadly. Yet importantly, as a collective, this issue emphasizes the many research gaps that remain. Exercise intervention studies are largely heterogeneous by nature, with significant variation in type, duration, frequency and intensity as well as in their overall reach, penetration and compliance. Reporting quality across studies introduces variation in the identification of particular components of exercise that contribute to positive outcomes. Taken together, this contributes to ambiguity in the field and limits translation and implementation of clinical recommendations. This issue highlights the critical need for further high quality, robustly designed research with transparent reporting in this area of exercise physiology.

## Author Contributions

CH and TM drafted the manuscript, with input from ALH. All authors approved the final version.

## Conflict of Interest

The authors declare that the research was conducted in the absence of any commercial or financial relationships that could be construed as a potential conflict of interest.
